# Nomogram to Predict Intensive Care Following Gastrectomy for Gastric Cancer: A Useful Clinical Tool to Guide the Decision-Making of Intensive Care Unit Admission

**DOI:** 10.3389/fonc.2021.641124

**Published:** 2022-01-11

**Authors:** Tao Pan, Xiao-long Chen, Kai Liu, Bo-qiang Peng, Wei-han Zhang, Meng-hua Yan, Rui Ge, Lin-yong Zhao, Kun Yang, Xin-zu Chen, Jian-kun Hu

**Affiliations:** Department of Gastrointestinal Surgery and Laboratory of Gastric Cancer, State Key Laboratory of Biotherapy, West China Hospital, Sichuan University and Collaborative Innovation Center for Biotherapy, Chengdu, China

**Keywords:** gastric cancer, intensive care medicine, resource allocation, complications, scoring system

## Abstract

**Background:**

We aimed to generate and validate a nomogram to predict patients most likely to require intensive care unit (ICU) admission following gastric cancer surgery to improve postoperative outcomes and optimize the allocation of medical resources.

**Methods:**

We retrospectively analyzed 3,468 patients who underwent gastrectomy for gastric cancer from January 2009 to June 2018. Here, 70.0% of the patients were randomly assigned to the training cohort, and 30.0% were assigned to the validation cohort. Least absolute shrinkage and selection operator (LASSO) method was performed to screen out risk factors for ICU-specific care using the training cohort. Then, based on the results of LASSO regression analysis, multivariable logistic regression analysis was performed to establish the prediction nomogram. The calibration and discrimination of the nomogram were evaluated in the training cohort and validated in the validation cohort. Finally, the clinical usefulness was determined by decision curve analysis (DCA).

**Results:**

Age, the American Society of Anesthesiologists (ASA) score, chronic pulmonary disease, heart disease, hypertension, combined organ resection, and preoperative and/or intraoperative blood transfusions were selected for the model. The concordance index (C-index) of the model was 0.843 in the training cohort and 0.831 in the validation cohort. The calibration curves of the ICU-specific care risk nomogram suggested great agreement in both training and validation cohorts. The DCA showed that the nomogram was clinically useful.

**Conclusions:**

Age, ASA score, chronic pulmonary disease, heart disease, hypertension, combined organ resection, and preoperative and/or intraoperative blood transfusions were identified as risk factors for ICU-specific care after gastric surgery. A clinically friendly model was generated to identify those most likely to require intensive care.

## Introduction

Intensive care units (ICUs) provide a limited number of specialized medical services and consume a significant portion of hospital resources for a minority of patients (1). Triage of high-risk surgical patients to ICUs may impact the outcomes of those with the highest probability of postoperative complications and deaths (2). However, in many hospitals, the availability of ICU is often limited (3), which may lead to canceled surgeries, delayed patient transfers (4), and increased morbidity and costs (5). Besides, severe acute respiratory syndrome coronavirus 2 (SARS-CoV-2) infection continues to grow across the world, and it is estimated that approximately 15% of patients presenting with SARS-CoV-2 will require ICU admission based on studies from Italy and China (6, 7). Therefore, identifying postoperative patients who need to be admitted to an ICU is a challenging but necessary task, especially during the coronavirus disease 2019 (COVID-19) pandemic.

Gastric cancer is the fifth most common cancer and the third leading cause of cancer-related death worldwide (8). Gastrectomy with curative intent is the most powerful treatment strategy to improve prognosis (9). Despite the advances in surgical and anesthetic techniques over the last decade, gastrectomy is associated with a high postoperative complication rate, ranging from 10.5% to 40.1% (10–12). Many complications require interventions and management that can be provided only in an ICU. As the frequency of elderly gastric cancer patients with more preexisting comorbidities is increasing (13, 14), the number of patients requiring ICU-specific care may inevitably increase. However, for many patients who will be undergoing gastrectomy for gastric cancer, postoperative admission to an ICU is only planned for surveillance purposes. ICU admissions for surveillance is not cost-effective and could lead to overuse of ICU resources (15). Furthermore, inappropriate ICU admission may be responsible for nosocomial infections and delirium (16). So, surgeons, anesthesiologists, and intensivists must identify which patients are most likely to require ICU-specific care by the end of surgery. Risk factors for postoperative ICU admission have been identified for several surgeries, including colon cancer surgery (17), lung resection (15), and total joint arthroplasty (18). Unfortunately, there are no studies that can guide the clinical decision-making of ICU admission after gastric cancer surgery.

Thus, we undertook this study to identify risk factors for ICU-specific care following gastrectomy for gastric cancer. We chose to evaluate preoperative and intraoperative factors because such a model would be more clinically friendly and useful than models based on postoperative complications or factors when ICU-specific admission would be inevitable and imminent. We aimed to use the risk factors to generate a nomogram to identify patients most likely to require ICU-specific care with the goal to provide a tool for optimizing the allocation of health care resources and ultimately improve postoperative outcomes.

## Methods

### Study Population and Ethical Issues

A total of 3,468 gastric cancer patients who underwent gastrectomy from January 2009 to June 2018 were included in the study. The inclusion criteria were as follows: 1) histologically confirmed gastric cancer; 2) patients underwent gastrectomy with radical or palliative intent. The exclusion criteria were as follows: 1) gastroenterostomy or exploration; 2) the gastric stump cancer; 3) with emergency surgery; 4) with incomplete medical data. The data of the patients were retrospectively extracted from the database of Surgical Gastric Cancer Patient Registry in West China Hospital under the registration number: WCH-SGCPR-2020-5. The establishment of this database was authorized by the Research Ethics Committee of West China Hospital. Due to the retrospective nature of the study, informed patient consent was waived. However, patient records were de-identified and anonymized before analysis.

### Clinicopathological Materials

Various preoperative and intraoperative variables were retrieved for risk factor selection: age, sex, body mass index (BMI), history of smoking, history of alcoholism, preoperative hemoglobin, preoperative albumin, the American Society of Anesthesiologists (ASA) score, preexisting comorbidities (including chronic pulmonary disease, heart disease, hypertension, diabetes mellitus, liver dysfunction), previous abdominal surgery, neoadjuvant chemotherapy, clinical TNM stage, the extent of surgery (curative gastrectomy or palliative gastrectomy), surgical approach, surgical procedure, reconstruction method, the extent of lymphadenectomy, number of retrieved lymph nodes, combined organ resection, surgery duration, tumor size, macroscopic type, and preoperative and/or intraoperative blood transfusions.

The ASA score was obtained from the anesthesia record sheet and had been determined by the anesthesiologist providing operating room care. The diagnosis of chronic pulmonary disease, heart disease, hypertension, diabetes mellitus, and liver dysfunction were made by physicians and recorded in the patient’s chart. Chronic pulmonary disease included any of the following diseases: chronic obstructive pulmonary disease, asthma, chronic bronchitis, bronchiectasis, emphysema, and occupational lung diseases (19). Heart diseases included any of the following diseases: arrhythmias, hypertensive heart disease, ischemic heart disease, valvulopathies, and heart failure (20). Hypertension was diagnosed according to the hypertension guideline (21). Blood transfusion was administration of packed red blood cells. The indication for blood transfusion was hemoglobin level <80 g/L. For patients with hemoglobin level between 80 and 100 g/L, blood transfusion was adopted based on the risk factors associated with hemodynamic instability and inappropriate oxygenation (22).

### Surgical Technique

The surgery was performed by experienced surgeons according to the Japanese gastric cancer treatment guidelines (23, 24). Intraoperative frozen section was routinely performed. Curative gastrectomy included cases in which an R0 resection was performed. Palliative gastrectomy was adopted only for patients with distant metastases but serious complications of gastric cancer (such as massive bleeding or pyloric obstruction) or for patients with residual tumor (R1 or R2 resections).

Combined organ resection was selectively performed for the purpose of curative resection or for patients with other comorbidities (such as cholecystectomy for gallbladder stone).

### Definition of Postoperative Intensive Care Unit-Specific Care

According to previous studies (15, 25), postoperative ICU-specific care was defined as the presence of one or more of the following characteristics: myocardial infarction, acute respiratory failure, shock, arrhythmia with hemodynamic instability, use of a variety of vasoactive drugs, reintubation, and maintenance of controlled ventilation longer than 48 h.

So, the ICU-specific group consisted of three groups of patients: 1) ICU treatment group: patients who were admitted to an ICU immediately after surgery and met the criteria of ICU-specific care; 2) Ward-ICU group: patients who were not admitted to ICU immediately after surgery but were admitted for an emergent reason, such as sudden cardiac arrest, acute respiratory failure, and any other situations that required ICU-specific care; 3) Refuse transfer group: patients who were admitted to the general ward after surgery and developed complications that required ICU-specific care; however, they refused to transfer to an ICU.

**Figure 1:**
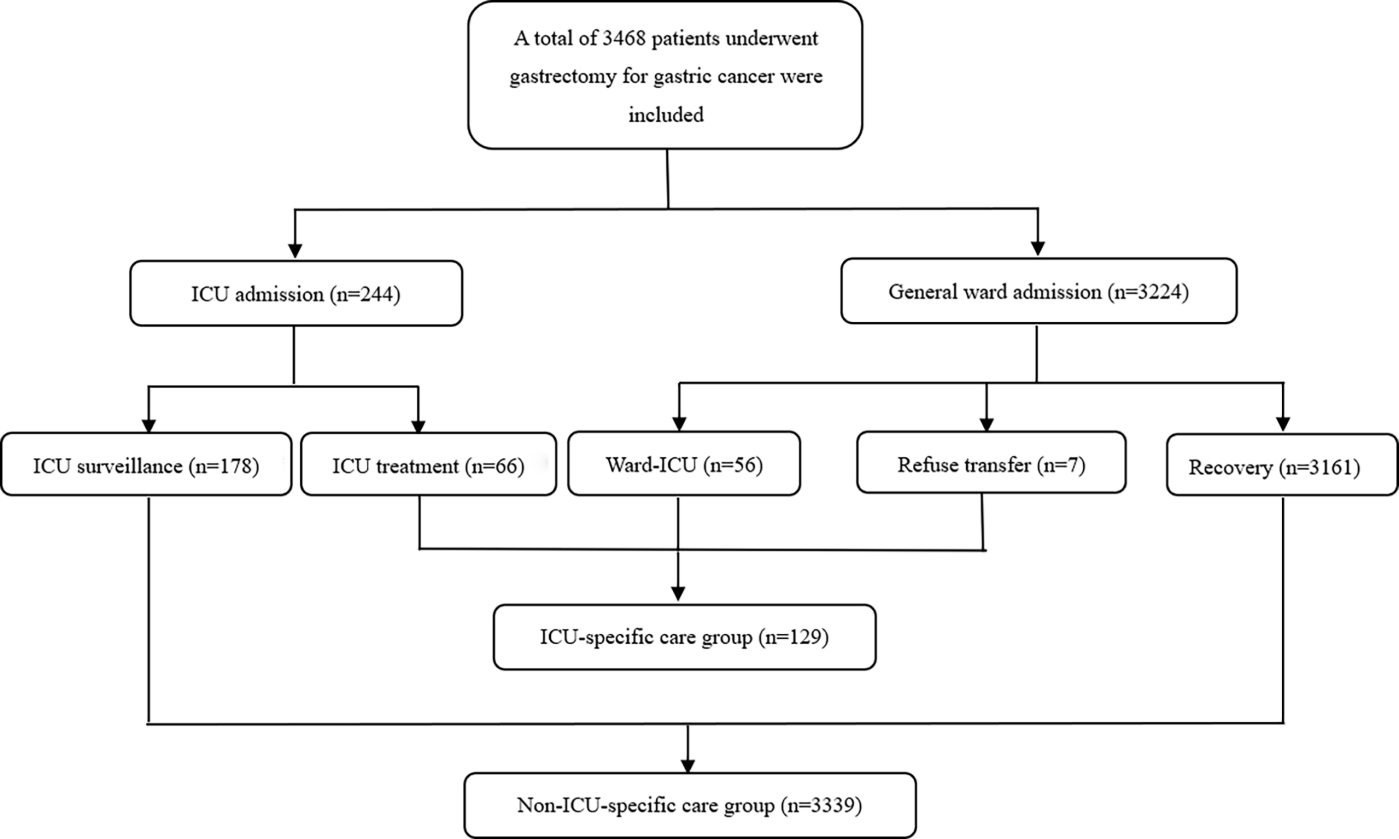
Patients’ flowchart. ICU, intensive care unit.

The Non-ICU-specific group consisted of two groups of patients: 1) ICU surveillance group: patients who were admitted to an ICU immediately after surgery for surveillance purposes and did not meet the criteria of ICU-specific care; 2) Recovery group: patients who were transferred to the general ward after surgery and then discharged without any complications. The patient flowchart is indicated in **Figure 1**.

### Statistical Analysis

Statistical analysis was conducted using R software (Version 3.6.1; https://www.r-project.org) and SPSS 20.0 (SPSS^®^, Chicago, IL, USA). Categorical variables are represented by number and percentage, while continuous variables are represented by mean ± standard deviation. We randomly assigned 70.0% of the patients to the training cohort and 30.0% to the validation cohort. We used the least absolute shrinkage and selection operator (LASSO) method to screen out the optimal variables with non-zero coefficients as risk factors (26). Then, based on the results of LASSO regression analysis, multivariable logistic regression analysis was used to establish the predictive model, and nomogram was further generated (27, 28). The predictive efficiency of the nomogram was evaluated by Harrell’s concordance index (C-index). Calibration curves were plotted to assess the calibration of the nomogram in both training cohort and validation cohort. A decision curve analysis (DCA) was also generated to determine the clinical usefulness of the nomogram. A p value <0.05 was considered to be statistically significant.

## Results

### Baseline Characteristics

A total of 3,468 gastric cancer patients who underwent gastrectomy from January 2009 to June 2018 were included in the study. There were 129 patients (3.7%) in the ICU-specific care group and 3,335 patients (96.3%) in the Non-ICU-specific care group (**Figure 1**). All patients were randomly divided into the training cohort (n = 2,428, 70.0% of the total patients) and the validation cohort (n = 1,040, 30.0% of the total patients). The characteristics of patients in the training and validation cohorts are shown in **Table 1**. There was no significant difference in any of the variables between the training and validation cohorts (all p > 0.05), indicating that the baseline was balanced between them.

**Table 1:** Characteristics of patients in the training and validation cohorts.

Variables		Training Cohort (n = 2,428)	Validation Cohort (n = 1,040)	p^†^
Age*	Year	58.2 ± 11.3	58.6 ± 11.3	0.463^‡^
Sex	Male	1,666 (68.6%)	734 (70.6%)	0.252
	Female	762 (31.4%)	306 (29.4%)	
BMI*	kg/m^2^	22.1 ± 2.9	22.2 ± 2.9	0.225‡
History of smoking	Without	1,512 (62.3%)	621 (59.7%)	0.155
	With	916 (37.7%)	419 (40.3%)	
History of alcoholism	Without	1,834 (75.5%)	769 (73.9%)	0.320
	With	594 (24.5%)	271 (26.1%)	
Preoperative hemoglobin	g/l	123.1 ± 25.1	122.5 ± 24.9	0.312
Preoperative albumin	g/l	41.5 ± 4.7	41.3 ± 4.8	0.513
ASA Score	1	145 (6.0%)	67 (6.4%)	0.760
	2	1,945 (80.1%)	819 (78.8%)	
	3	337 (13.9%)	153 (14.7%)	
	4	1 (0)	1 (0.1%)	
Chronic pulmonary disease	Without	1,991 (82.0%)	871 (83.8%)	0.214
	With	437 (18.0%)	169 (16.2%)	
Heart disease	Without	2,345 (96.6%)	1,009 (97.0%)	0.508
	With	83 (3.4%)	31 (3.0%)	
Hypertension	Without	2,223 (91.6%)	953 (91.6%)	0.940
	With	205 (8.4%)	87 (8.4%)	
Diabetes mellitus	Without	2,331 (96.0%)	1,003 (96.4%)	0.540
	With	97 (4.0%)	37 (3.6%)	
Liver dysfunction	Without	2,250 (92.8%)	959 (92.2%)	0.639
	With	178 (7.2%)	81 (7.8%)	
Previous abdominal surgery	Without	1,997 (82.2%)	863 (83.0%)	0.603
	With	431 (17.8%)	177 (17.0%)	
Neoadjuvant chemotherapy	Without	2,396 (98.7%)	1,026 (98.7%)	0.947
	With	32 (1.3%)	14 (1.3%)	
Clinical T Stage	T0/1/2	981 (40.4%)	449 (43.2%)	0.129
	T3/4	1,447 (59.6%)	591 (56.8%)	
Clinical N Stage	Negative	1,146 (47.2%)	509 (48.9%)	0.346
	Positive	1,282 (52.8%)	531 (51.1%)	
Distant metastases	Without	2,250 (92.7%)	958 (92.1%)	0.571
	With	178 (7.3%)	82 (7.9%)	
Surgical approach	Open	2,074 (85.4%)	895 (86.1%)	0.624
	Laparoscopic	354 (14.6%)	145 (13.9%)	
Extent of surgery	Radical	2,177 (89.7%)	926 (89.0%)	0.583
	Palliative	251 (10.3%)	114 (11.0%)	
Surgical procedure	Distal gastrectomy	1,453 (59.8%)	604 (58.1%)	0.429
	Proximal gastrectomy	245 (10.1%)	119 (11.4%)	
	Total gastrectomy	730 (30.1%)	317 (30.5%)	
Reconstruction method	Billroth-1	330 (13.6%)	143 (13.8%)	0.699
	Billroth-2	1,062 (43.7%)	441 (42.4%)	
	Roux-en-Y	789 (32.5%)	337 (32.4%)	
	Esophagogastrostomy	247 (10.2%)	119 (11.4%)	
Extent of lymphadenectomy	D1/D1+	396 (16.3%)	168 (16.2%)	0.909
	D2/D2+	2,032 (83.7%)	872 (83.8%)	
Number of retrieved lymph nodes*	–	30.0 ± 13.7	30.4 ± 14.1	0.388^‡^
Combined organ resection	Without	2,279 (93.9%)	977 (93.9%)	0.929
	With	149 (6.1%)	63 (6.1%)	
Surgery duration*	Minute	230.5±44.8	230.0±45.5	0.726‡
Tumor size*	Cm	5.2±3.0	5.3±3.0	0.359‡
Macroscopic type	Early Gastric Cancer	483 (19.9%)	221 (21.3%)	0.177
	Borrmann-1	41 (1.7%)	20 (1.9%)	
	Borrmann-2	880 (36.2%)	368 (35.4%)	
	Borrmann-3	852 (35.1%)	379 (36.4%)	
	Borrmann-4	172 (7.1%)	52 (5.0%)	
Preoperative and/or intraoperative blood transfusion	Without	2,182 (89.9%)	941 (90.5%)	0.581
	With	246 (10.1%)	99 (9.5%)	
ICU-specific care	Without	2,334 (96.1%)	1,005 (96.6%)	0.540
	With	94 (3.9%)	35 (3.4%)	

Values in parentheses are percentages unless indicated otherwise; *values are mean ± standard deviation. ^†^χ^2^ test, except ^‡^paired t test.

BMI, body mass index; ASA, American Society of Anesthesiologists; ICU, intensive care unit.

### Risk Factor Selection

**Figure 2:**
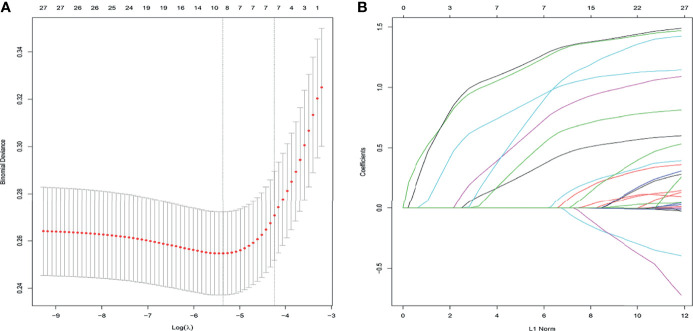
Clinicopathological features selection using the LASSO logistic regression model. Final predictors include age, the ASA score, chronic pulmonary disease, heart disease, combined organ resection, and preoperative and/or intraoperative blood transfusion. **(A)** Optimal parameter (λ) selection in the LASSO model used 5-fold cross-validation and minimum criteria. The partial likelihood deviance (binomial deviance) curve was plotted vs. log(λ). Dotted vertical lines were drawn at the optimal values by using the minimum criteria and the 1 SE of the minimum criteria (the 1-SE criteria). **(B)** LASSO coefficient profiles of the 27 features. A coefficient profile plot was plotted against the log(λ) sequence, and the 7 non-zero coefficients were chosen at the values selected using 5-fold cross-validation. ASA, American Society of Anesthesiologists; LASSO, least absolute shrinkage and selection operator; SE, standard error.

We performed a LASSO regression analysis to evaluate the 29 variables in the training cohort (**Figure 2**). Finally, we retained 7 variables with non-zero coefficients as potential predictors of the prediction model. These predictors included age, the ASA score, chronic pulmonary disease, heart disease, hypertension, combined organ resection, and preoperative and/or intraoperative blood transfusions.

### Nomogram and Validation

**Figure 3:**
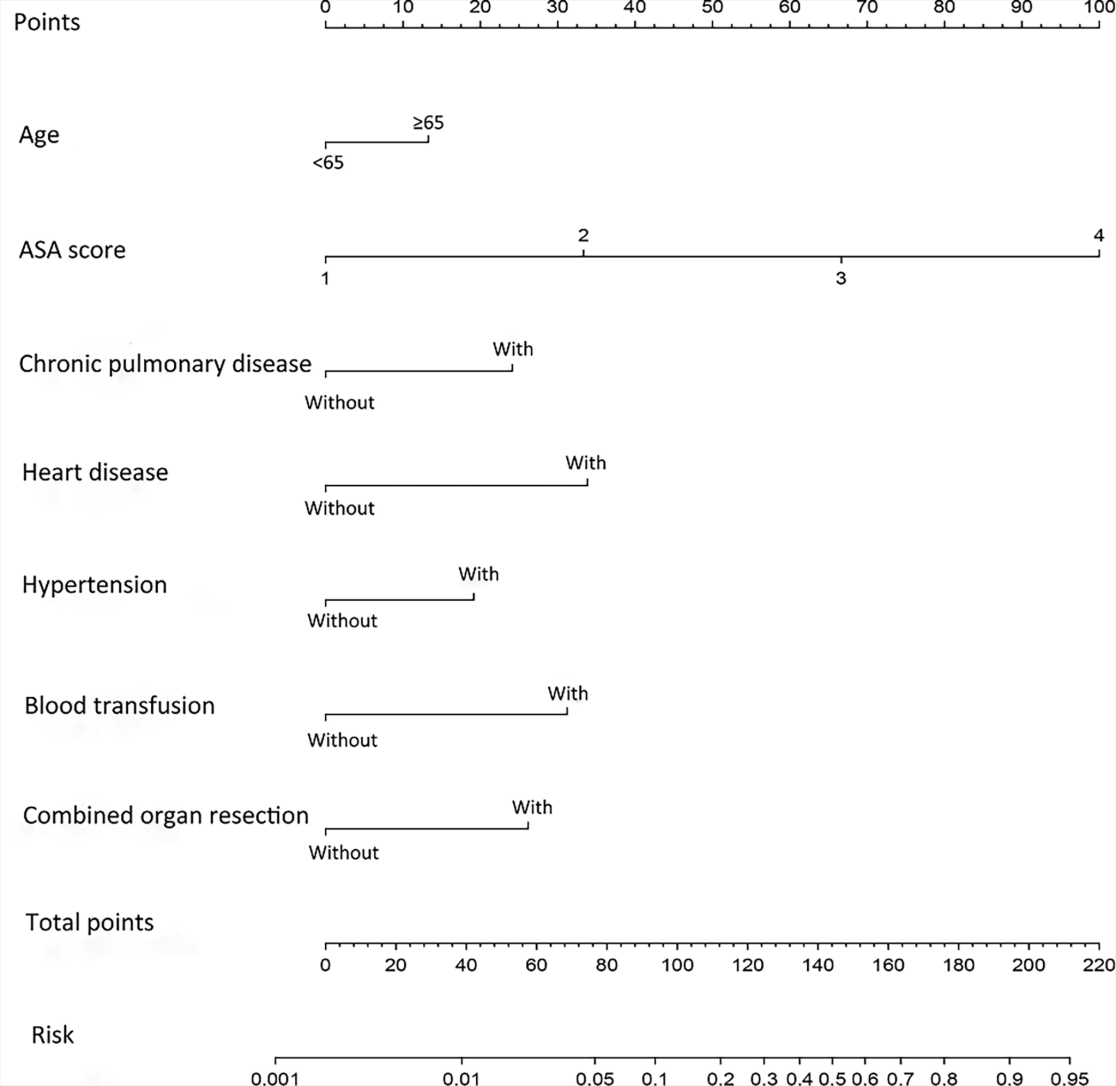
Nomogram for predicting ICU-specific care following gastrectomy for gastric cancer. The prediction nomogram was developed in the training cohort, with age, ASA score, chronic pulmonary disease, hypertension, combined organ resection, and preoperative and/or intraoperative blood transfusions incorporated. ASA, American Society of Anesthesiologists; ICU, intensive care unit. Blood transfusion, preoperative and/or intraoperative blood transfusions.

**Figure 4:**
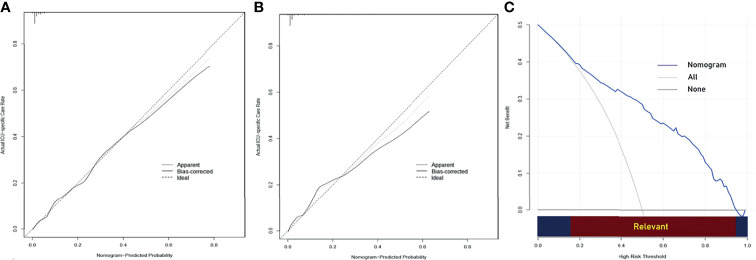
**(A)** Calibration curve of the nomogram for predicting ICU-specific care following gastrectomy for gastric cancer in the training cohort. **(B)** Calibration curve of the nomogram for predicting ICU-specific care following gastrectomy for gastric cancer in the validation cohort. **(C)** Decision curve analysis (DCA) for predicting ICU-specific care following gastrectomy for gastric cancer. The y-axis represents net benefit. The x-axis shows the threshold probability. “All” refers to the assumption that all patients need ICU-specific care, and “None” refers to the assumption that no patient needs ICU-specific care. When the score is within the range 0.14–0.95 (Relevant), using the nomogram to predict ICU-specific care adds more net benefit than the treat-all or treat-none strategies. ICU, intensive care unit.

To get a more comprehensive view of the relationship between the need for ICU-specific care and these predictors, we further performed a multivariable logistic regression analysis and constructed a predictive model. The results of the logistic regression analysis were given in **Table 2** and visualized in the form of a nomogram plot to help practice in the clinic (**Figure 3**). The C-index of the model was 0.843 in the training cohort and 0.831 in the validation cohort. The calibration curves of the ICU-specific care risk nomogram suggested great agreement in both training cohort and validation cohort (**Figures 4A, B**).

**Table 2:** Risk factors for ICU-specific care following gastrectomy for gastric cancer.

Risk Factors	β	Odds Ratio (95% CI)	p
Age (vs. <65 years old)	0.587	1.798 (1.104–2.928)	0.018
ASA Score (vs. 1)			
2	1.060	2.888 (0.389–21.421)	0.300
3 and 4	2.536	12.624 (1.683–94.677)	0.014
Chronic pulmonary disease (vs. without)	1.065	2.900 (1.799–4.675)	<0.001
Heart disease (vs. without)	1.474	4.366 (2.258–8.442)	<0.001
Hypertension (vs. without)	0.831	2.295 (1.305–4.037)	0.004
Combined organ resection (vs. without)	1.394	4.031 (2.143–7.582)	<0.001
Preoperative and/or intraoperative blood transfusions (vs. without)	1.128	3.091 (1.864–5.125)	<0.001

β is the regression coefficient. ASA score was entered into the logistic model by combining the patients with ASA score = 3 and those with ASA score = 4 because of the limited number of patients with ASA score = 4 in the total population (n = 1 in the training cohort; n = 1 in the validation cohort).

SA, American Society of Anesthesiologists; CI, confidence interval; ICU, intensive care unit.

### Clinical Usefulness

The DCA for the predictive nomogram is shown in **Figure 4C**. The analysis indicated that using the nomogram to predict ICU-specific care following gastrectomy for gastric cancer added more net benefit than the treat-all or treat-none strategies when the threshold probability was within the range 0.14–0.95.

## Discussion

This study showed that a small (3.7%) but important proportion of patients required ICU-specific care following gastrectomy for gastric cancer. These patients tended to be older and more likely to have a higher ASA score, chronic pulmonary disease, heart disease, hypertension, combined organ resection, and blood transfusion before and/or during surgery. Recent data have shown that ICU admission after surgery only for surveillance purposes may increase medical costs without the expected additional benefits for patients (29, 30). Therefore, identifying patients at a high risk of postoperative ICU-specific care can help improve postoperative outcomes and optimize the allocation of health care resources, especially during the COVID-19 pandemic. To our knowledge, this was the first study that can guide the clinical decision-making of ICU admission after gastrectomy for gastric cancer. The model can be used to evaluate ICU resource allocation by retrospectively identifying patient groups whose characteristics indicate that they may not have needed ICU admission. The ability to identify low-risk admission patients allows managers to implement protocols and educational programs for providing effective and safe care alternatives in intermediate care units or general wards.

In the present study, older age was identified as a risk factor for postoperative ICU-specific care. Multiple previous studies have demonstrated that older age was independently associated with postoperative complications after gastrectomy (31, 32). Some complications can be managed only in an ICU. Although the incidence of gastric cancer has been declining due to longer life expectancy, the number of aged patients with this disease is continuously increasing (13). So, we can foresee that an increasing number of patients may require ICU-specific care after gastrectomy for gastric cancer.

We also found that several preexisting comorbidities were also associated with postoperative ICU-specific care, such as chronic pulmonary disease, heart disease, and hypertension. All these factors have been identified as risk factors for postoperative morbidity and mortality after gastrectomy for gastric cancer in previous studies (33–37). So, special attention should be paid to patients with these comorbidities, and we believe that prior treatment of these preoperative comorbidities is essential to the postoperative recovery of patients with gastric cancer.

The ASA score was found to have a strong influence on ICU-specific care in the present study. Several studies have reported that it was a risk factor for ICU admission following other surgeries (38, 39). The ASA score has the advantages of simplicity and universality (40) and is an effective risk indicator whether used alone or in combination with other parameters (41). A difficulty in using it in patient assessment is the limited interobserver reliability (42). However, a previous study has confirmed that the ASA score had the greatest validity and highest interobserver reliability when assigned by the responsible anesthesiologist in the operating theater (43). Therefore, we obtained the ASA score from the anesthesiologist chart and had been determined by the anesthesiologist providing operating theater care to maximize its validity and reliability.

Among all the surgical factors, only combined organ resection was identified as a risk factor for ICU-specific care in our study. These findings were supported by a previous study (44), which demonstrated that combined organ resection had an increased risk for postoperative complications and mortality. Our study did not identify any association between surgical approach (open or laparoscopic), surgical procedure (distal, proximal, or total gastrectomy), extent of surgery (radical or palliative), or extent of lymphadenectomy (D1/D1+ or D2/D2+) and postoperative ICU-specific care. Laparoscopic gastrectomy has gained popularity in the treatment of gastric cancer in China, Japan, and Korea (45). Multiple randomized trials have demonstrated that there was no significant difference in postoperative complications and deaths between laparoscopic and open gastrectomy for patients with preoperative stage I gastric cancer (12) and for patients with advanced gastric cancer who underwent distal gastrectomy (45). In terms of surgical procedure, previous studies reported mixed results. Shin et al. (46) reported that surgical procedure was not associated with postoperative complications. However, Lee et al. (34) reported that total gastrectomy was an independent risk factor for postoperative complications. In the present study, the extent of surgery (radical vs. palliative resection) was not identified as a risk factor for ICU-specific care. In a previous study (47), there was no significant difference in mortality and morbidity rate after palliative or radical surgery. The possible explanation is that patients undergoing palliative surgery may be in poorer general condition, but the surgery is less invasive and shorter in duration (47). In terms of the extent of lymphadenectomy, mortality and morbidity rates did not differ significantly between D1 and D2 group whether in retrospective (48) or prospective studies (11). In our personal opinion, D2 lymph node dissection can be safely performed by senior gastric cancer surgeons.

In the present study, blood transfusion was also found to be a risk factor for ICU-specific care. These findings were in accordance with a previous study (49). There is a high incidence of anemia in patients with advanced gastric cancer (50). In addition, gastrectomy with lymphadenectomy sometimes leads to excessive bleeding even performed by experienced surgeons (51). Thus, perioperative blood transfusion is sometimes inevitable when performing gastrectomy for advanced gastric cancer. Although blood transfusion can be lifesaving for gastric cancer patients with severe anemia by improving their oxygen delivery capacity and tissue perfusion, it can also result in systemic inflammation and other transfusion-related adverse events, especially acute lung injury and infection (52, 53). Furthermore, preoperative and intraoperative blood transfusions may reflect the patient’s poor systemic condition or complexity of the surgery (54). So, special attention should be paid to patients who have blood transfusion in the perioperative period.

The endpoint of our study was postoperative ICU-specific care. However, postoperative ICU-specific care has been defined differently in previous studies. Two studies (29, 30) defined at least 24 h in an ICU setting as postoperative ICU-specific care, regardless whether the patients received any active life-supporting treatments (1) or not. Dahm et al. (25) defined ICU-specific care as the presence of one or more of the following characteristics: mechanical ventilation longer than 12 h, continuous intravenous infusion of vasoactive medication, or a postoperative event mandating treatment in an ICU setting (pulmonary embolism, myocardial infarction, or arrhythmia with hemodynamic instability). Kim et al. (15) defined ICU-specific care as the presence of one or more of the following characteristics: reintubation, maintenance of controlled ventilation, hemodynamic instability, shock, acute respiratory failure, use of multiple vasoactive drugs, and cardiac arrhythmia. Patients who were admitted to the ICU and then transferred to the general ward the day after the surgery were deemed as non-specific care group in their study. In the present study, we defined ICU-specific care group as the presence of one or more of the following characteristics: myocardial infarction, acute respiratory failure, shock, arrhythmia with hemodynamic instability, use of a variety of vasoactive drugs, reintubation, and maintenance of controlled ventilation longer than 48 h. This parameter was based on our institutional guidelines that patients in the postoperative ICU are expected to be extubated within 48 h. We also included several life-supporting treatments that are best or unique to performed in an ICU setting. Such a definition may be more comprehensive and clinically relevant (55).

In the present study, we constructed a nomogram to guide the clinical decision-making of ICU admission. Medical providers could make individualized predictions of the probability of receiving intensive care with this easy-to-use model, which is in accordance with the current trend toward personalized medicine (56). Improved health care resource use and reduced costs might be achieved by providing care for these patients in general wards or intermediate care units, especially during the COVID-19 pandemic. The most important argument for the use of the nomogram is based on the need to interpret a patient’s need for additional treatment or care. However, discrimination and calibration cannot capture the clinical consequences of specific levels of discrimination or degrees of miscalibration (57). The DCA showed that using the nomogram to predict the probability of receiving intensive care is more beneficial than the treat-all or the treat-none strategies if the threshold probability of an individual is within 0.14–0.95.

The strengths of the study were that it included a wide range of variables with ICU-specific care from a large cohort. The proposed prediction nomogram was generated based on routinely collected preoperative and intraoperative data to maximize its application and ensure its generalizability. The study also had some limitations. First, the study was conducted retrospectively; there may have been some inherent selection biases. A prospective study should be carried out to validate the prediction model. Second, our study was a monocentric study and the results were validated only internally; further external validation should be performed to make sure whether these results could be applied to other institutions.

## Conclusions

Several risk factors for ICU-specific care after gastrectomy for gastric cancer were identified. A clinically friendly model with excellent ability was generated to identify those most likely to require intensive care.

## Data Availability Statement

The raw data supporting the conclusions of this article will be made available by the authors without undue reservation.

## Ethics Statement

This study was based on the information gathered from the database of the Surgical Gastric Cancer Patient Registry of West China Hospital (WCH-SGCPR) under registration number WCH-SGCPR-2020-5. The establishment of this database was approved by the Research Ethics Committee of West China Hospital. Informed patient consent was waived because of the retrospective nature of the analysis.

## Author Contributions

J-KH, TP, and X-LC. Administrative support: J-KH, TP, and X-LC. Provision of study materials or patients: KL, W-HZ, RG, M-HY, L-YZ, KY, and X-ZC. Collection and assembly of data: TP, B-QP and X-LC. Data analysis and interpretation: TP and X-LC. Article writing: All authors. Final approval of article: All authors. All authors contributed to the article and approved the submitted version.

## Funding

This study was funded by the Foundation of Science & Technology Department of Sichuan Province (2019YFS0255); the 1.3.5 project for disciplines of excellence, West China Hospital, Sichuan University (No. ZY2017304); and the Sichuan Province Cadre Health Care Research Project (No. 2017-114).

## Conflict of Interest

The authors declare that the research was conducted in the absence of any commercial or financial relationships that could be construed as a potential conflict of interest.

## Publisher’s Note

All claims expressed in this article are solely those of the authors and do not necessarily represent those of their affiliated organizations, or those of the publisher, the editors and the reviewers. Any product that may be evaluated in this article, or claim that may be made by its manufacturer, is not guaranteed or endorsed by the publisher.
